# Annotations

**Published:** 1891-05-23

**Authors:** 


### Annotations.

**A Jewish Lady Centenarian.**

There died last week at St. John's Wood, Mrs. Hart, widow of the late Mr. Joel Hart, one of the founders of the Jews' Hospital at Norwood. Mrs. Hart had reached the extaordinary age of 106 years, and had remained in the almost undiminished possession of all her faculties until within a very short period of her death. She retained full consciousness until the last hour. It is exceedingly interesting to find a person of this unusual age about the length of whose years there cannot be the slightest doubt. But certain other facts in this ancient lady's history are still more interesting and striking. She was the parent of five succeeding generations, and at the time of her death she could count her direct descendants to the number of upwards of five hundred persons. Several of these were great-great-grandchildren. As showing the extraordinary vigour of the old lady's constitution it may be mentioned that she had an attack of bronchitis at the age of 103, from which she made a perfect recovery. All these facts we have from a member of the Jewish community who knew Mrs. Hart intimately. It would have been still more interesting if one could have obtained particulars of the longevity of her parents and her near relatives; and also as to her habits and mode of life. Had she always lived in London? Was she a total abstainer? Was she of robust build? Was she of calm and placid temper? These and many other similar facts would greatly interest both aged people who intend to be centenarians, and younger persons who look with kindly veneration upon those who have sustained the world's shocks and battles for so many years. Our readers, like ourselves, will be grateful to the correspondent who has furnished us with the interesting particulars we have been able to give about Mrs. Hart. We know of few things which will give more pleasure to the middle-aged and the old than the record of facts and experiences of this nature.

**Weather Science at the House of Commons.**

It is announced this week that more than ninety Members of Parliament are now suffering, or have quite lately suffered, from influenza. Certain persons, who think they know, affirm that this is all nonsense, and that many of the honourable Members are merely suffering from severe colds arising from the chilly and changeable atmosphere of the House of Commons. It is stated that, such is the wretched level of practical scientific knowledge which a legislative assembly that includes Sir Henry Roscoe possesses, that no attempt whatever is made to keep the temperature of the House at anything like a uniform level. On the contrary, it is declared to be notorious that the man-servant who has charge of the fires of the House lights those fires when he pleases, and leaves them unlighted when he pleases, and does, in fact, exactly what his own unchallenged discretion or indiscretion prompts him to do. The result of this is the disablement and severe suffering of more than ninety legislators, distinguished and otherwise. It is highly probable that not more than nine of those ninety gentlemen have suffered from true infectious influenza. The other eighty have had more or less severe colds due to the varying temperature of the chamber in which they sit and the varying moistness of its atmosphere. Now, to anybody who understands the merest elements of physiology this is imbecility *in excelsis*. All the legislators of this great country are willing to place their lives and health, in a most fickle climate and at a most dangerous season of the year, entirely at the mercy of an uninstructed and possibly indolent man-servant. It is incredible, and yet it is true. Mr. Gladstone and Mr. Smith, Mr. Balfour and Mr. Morley, Dr. Cameron and Sir Walter Foster, all men of practical sense and scientific attainments, some of them with many years of medical experience, have acquiesced in this imbecile and inconsequent compact. No words can adequately represent the ridiculousness of the situation. Probably the combined wages of the cooks of the honourable members would amount to not much less than £30,000 a-year. But the management of the English climate is a matter requiring infinitely more skill and judgment than the management of English cookery; and yet all these gentlemen leave to one man, whose wages may be represented by a limited number of shillings per week, the sole management of the atmosphere and climate in which half their working time is spent. Notwithstanding all this, we laud and magnify the scientific attainments of the present age. Can darkest Africa itself show practical scientific stupidity of a sublimer type?

**The Doctor's Pound of Tea.**

One step more, and certain doctors will have reached as deep a depth of professional turpitude as is possible to the efforts of mortal man. According to the *British Medical Journal*, there is at least one practitioner in the profession whose fee for attendance on a child is two shillings per annum, medicine included. This gentleman only needs to take the further step of giving a pound of tea per annum, as well as his professional services and his physic, to each child, in order to reach the ideally perfect level of "medical providence." Who the man may be we do not know: what he may be we know well! We have expressed here and elsewhere a certain amount of pity for the poor practitioner who, when burdened with a family, and reduced to sore domestic straits, has unwisely endeavoured to increase his practice and his income by advertising; but of the man who pretends to treat children medically and to supply them with medicine for two shillings a-year, nothing can be said which will sufficiently express the degradation into which he has fallen, and the contempt which he deserves. An illustration will show what we mean. If a person were to go about the country offering bona-fide golden sovereigns for two shillings a-piece, everybody would know what to think of him. He would stand confessed a madman or an impudent cheat. A doctor can no more undertake to attend an indefinite number of children and to give them the kind of medicine they require for two shillings a-year a head than the other man can afford to sell golden sovereigns for two shillings apiece. Everybody, therefore, who sees or hears of a medical man who makes this kind of offer should put a mark against his name. He is to be classed with baby farmers and child insurance murderers. Conscience he is destitute of; sense he cannot possess; cunning of the lowest, meanest, and most stupid kind is his distinguishing attribute. Everybody who employs him must be at least a foolish person and a dupe. If he be not foolish he is wicked, because he trusts the life and health of helpless children to the care of a man who is self-confessed a cheat. It ought to be possible to appeal to the medical profession with some hope of putting an effectual check upon "two shillings a-year" doctors. But there is neither unanimity of feeling nor mutual confidence and respect in the profession. Chaos, distrust, and dislike are everywhere manifest. But though the profession itself is so feebly ruled, so blindly guided, and so universally disloyal, there is no reason why the public should not protect itself against medical cheats who practice their knavery under the protection of legal qualifications. "Two shillings a-year" doctors, and the whole class of provident dispensary-mongers who live by their wits and fight for their own hands alone, are among the most dangerous and disreputable impostors of the present times. Every honest parent should shun them as he would shun a grocer who deals in short weights, or a butcher who is a purveyor of diseased meat. When will poor people learn that the doctor who works for less than "sweaters'" wages is bound to be a workman of the very miserablest class, and can hardly avoid being a scoundrel to boot?

